# Patient portals and health apps: Pitfalls, promises, and what one might learn from the other

**DOI:** 10.1016/j.hjdsi.2016.08.004

**Published:** 2016-10-03

**Authors:** Jessica L. Baldwin, Hardeep Singh, Dean F. Sittig, Traber Davis Giardina

**Affiliations:** a Houston Veterans Affairs Center for Innovations in Quality, Effectiveness and Safety, Michael E. DeBakey VA Medical Center, Houston, TX, USA; b Section of Health Services Research, Department of Medicine, Baylor College of Medicine, Houston, TX, USA; c School of Biomedical Informatics, University of Texas Health Science Center, Houston, TX, USA

## Abstract

Widespread use of health information technology (IT) could potentially increase patients’ access to their health information and facilitate future goals of advancing patient-centered care. Despite having increased access to their health data, patients do not always understand this information or its implications, and digital health data can be difficult to navigate when displayed in a small-format, complex interface. In this paper, we discuss two forms of patient-facing health IT tools—patient portals and applications (apps)—and highlight how, despite several limitations of each, combining high-yield features of mobile health (mHealth) apps with portals could increase patient engagement and self-management and be more effective than either of them alone. Patient portal adoption is variable, and due to design and interface limitations and health literacy issues, many people find the portal difficult to use. Conversely, apps have experienced rapid adoption and traditionally have more consumer-friendly features with easy log-in access, real-time tracking, and simplified data display. These features make the applications more intuitive and easy-to-use than patient portals. While apps have their own limitations and might serve different purposes, patient portals could adopt some high-yield features and functions of apps that lead to engagement success with patients. We thus suggest that to improve user experience with future portals, developers could look towards mHealth apps in design, function, and user interface. Adding new features to portals may improve their use and empower patients to track their overall health and disease states. Nevertheless, both these health IT tools should be subjected to rigorous evaluation to ensure they meet their potential in improving patient outcomes.

## 1. Introduction

There is growing interest in electronic access to health information and the use of digital data for both disease and health-related tracking. Widespread use of health information technology (IT) could potentially increase patients’ access to their health information and facilitate future goals of advancing patient-centered care.^1^ For example, health IT can be used to facilitate information exchange with clinicians and instruct patients when to act upon clinical issues, such as out of range physiologic parameters, follow-up of test results, and complications of medication use.^2^ Tools such as personal health records, patient portals, and various mobile health (mHealth) applications (apps) have been developed to help patients engage in their own care. Already, a significant number of patients use health IT; therefore, it is essential that patient-facing health IT be tailored to their needs. In this paper, we discuss two forms of patient-facing health IT tools—patient portals and apps—to highlight how, despite several limitations of each, combining high-yield features of mHealth apps with portals could increase patient engagement and self-management and be more effective than either of them alone. This could potentially improve both patient experience and outcomes related to patient-facing health IT.

Patient-facing health IT should be simply designed to encourage and sustain use and engage patients at various levels of health literacy.^3^ Patients increasingly express interest in being involved in medical decision-making and desire access to their health information.^4^ Despite having increased access to their health data, patients do not always understand this information or its implications, and digital health data can be difficult to navigate when displayed in a small-format, complex interface. For example, test results are not always displayed in a way that is easy for the patient to understand (e.g., with normal ranges clearly shown, along with implications of abnormal results). There is also little evidence that patient portal design addresses patients’ needs outside of meeting the “meaningful use” patient engagement criteria.^5^ It is imperative to keep patients’ needs in mind because patient-facing health IT users in the long run will not be just the early adopter health and technology “enthusiasts,” but regular people in need of better disease control and management.^6^

## 2. Emergence of patient-facing health IT

According to Pew Research, 7 in 10 U.S. adults say they track at least one health indicator.^7^ Although the number of patients interested in accessing their test results and tracking their health parameters has increased, patient portal use nationally is variable.^5,8^ For example, Athenahealth reports a 25% adoption rate across 1100 fee-for-service provider groups.^9^ Kaiser Permanente, an institution that has used portals for over a decade, reports that as of the third quarter of 2015, about 70% (5.2 million patients) of eligible adult members registered to use its My Health Manager patient portal.^10^ Group Health Cooperative (Seattle, WA) reports 73% of enrollees in Group Health Practices are registered and ID-verified to use its patient portal website as well as its mobile app, which offers the same constellation of services as the patient portal.^11^ However, registration rates and ID verifications do not account for the people who register but do not actively use the portal. Based on anecdotal data from a project we are conducting to determine patient preferences when viewing test results through portals, several patients have reported login issues and difficulty navigating portals.^12^

Conversely, the use of “easy to access” mHealth apps has increased dramatically over the past few years. Estimates suggest that by 2018, half of all smartphone and tablet users will have downloaded a mHealth app.^13^ The rise in health apps and health tracking software can be partially attributed to peoples’ growing interest in wearable devices and new applications that enable and engage patients to do more for their health care. Further, many apps rely heavily on social networking and the community experience allowing users to continuously track their activities and compare themselves with friends, family, and the larger community. Compared to most portals, apps appear to be more consumer-centric in design, and therefore, easier to use.

## 3. Pitfalls and promises of patient portals and health applications

As a secure online website providing patients access to their health information, the portal aims to improve quality of care by engaging patients as active participants in their care. While portal functions vary, most allow patients to view laboratory test results, immunizations, medications, and allergies, as well as to send secure messages to their physician.^14^ However, the portal can be difficult to navigate, and patients may struggle to understand their medical information. For instance, in our previous work we found that test result display and graphing were often confusing to patients, and they reported that portals were not user-friendly.^15^ A recent systematic review of patient and provider attitudes toward patient portal use found that the most negatively-perceived feature was user-friendliness, making the portal difficult to navigate.^16^ Our work exploring patient’s experiences using the portal to view test results echoes this finding, as many patients reported having difficulty locating their test results in the portal.^17^ When patients interact with their test results, they need to know the purpose of the test, the interpretation of the result, and next steps.^18^ Addressing these issues may help improve patient-centered care.

On the other hand and for a different engagement purpose, several companies have designed and created various tracking applications to encourage people to actively participate in their health. Applications, such as Mango Health (San Francisco, CA), Fitbit (San Francisco, CA), and Apple (Cupertino, CA) iPhone 6’s built in Health app, have consumer-friendly features with easy login access, real-time tracking, and simplified data display.^19^ From a patient standpoint, these features likely make the applications more intuitive and easy-to-use than patient portals. Furthermore, mHealth apps live on mobile devices, which make them easily accessible with little effort to login after setting up the account. This ubiquitous access is one of the reasons mobile technology is rapidly replacing desktop technologies.

Although apps might serve a different purpose, patient portals could adopt certain app features that lead to better engagement success with patients. Mobile apps have the capability to record several types of data, such as activity level, nutrition, and sleep, as well as data related to a consumer’s condition or disease, such as diabetes or asthma. For instance, Apple’s ResearchKit, although not designed as a health tracking application, offers several features that could be useful for health monitoring. It collects data and simultaneously encourages users to track their health by prompting daily health assessments. mHealth apps offer symptom management activities, which are not a standard feature universally available in patient portals.^16^ For example, LifeMap Solutions (San Jose, CA) has an application for Chronic Obstructive Pulmonary Disease management that provides medication reminders and tracks users’ symptoms to identify abrupt declines in their condition. Sentrian (Aliso Viejo, CA), a patient intelligence company, uses biosensors (i.e., blood glucose biosensor) to detect deteriorating health of patients to prevent avoidable hospitalizations.^20^ There are also apps that allow users to view their test results, such as Healthvana (Los Angeles, CA) and Labcorp (Research Triangle Park, NC). Healthvana, for instance, provides patients with interpretation of sexually transmitted infection results and follow-up instructions.^21^

While it is not clear if apps influence patient behavior, condition-specific apps may help patients improve outcomes.^22,23^ Nevertheless, mHealth apps’ features and functionality do not extend widely to provide users access to their institutionally-generated health data. At this point we also do not really know the value of the data generated by mHealth apps, and researchers are still determining how to best use the data from new apps like ResearchKit.^24,25^ These apps are also not heavily regulated and could contain poor quality or incorrect information, and some apps have been found to produce incorrect or inconsistent data.^26,27^ Despite increasing use of mHealth apps, up to 80% of apps are abandoned after only two weeks, suggesting more research is needed to understand what features engender longevity.^28^ Additionally, a recent study regarding health app use among vulnerable populations found that participants lacked confidence with the technology and expressed frustration with design and navigation. The authors called for participatory design, testing, and training with diverse patient populations to improve use.^29^

While mobile apps may offer more personalized interactions, it has been suggested that these apps need to be connected to personal health records to be effective and improve patient outcomes.^30^ However, there are legal concerns related to data protection and some uncertainty as to when and if mHealth apps fall under HIPAA or a developer’s own privacy policy, if available.^31–34^ Thus, additional research must examine and determine the usefulness, relevance, credibility, and accuracy of health apps, as well as how they are used by patients and health care professionals.^35^

## 4. Socio-technical comparison of patient portals and health applications

In this section, we compare and contrast apps and portals using a sociotechnical lens. In our previous work, we have used an eight-dimension socio-technical model to evaluate other health IT innovations,^36^ interventions,^37^ applications,^38^ and devices^39^ implemented within a health care system.^1^ In Table 1, we compare apps and portals along the eight dimensions.

## 5. A vision for better portals

Patient portals are intended to engage patients by giving them access to medical information; however, if patients are unable to understand the information or the system is not usable, patients will not take advantage of them. Despite several aforementioned drawbacks, apps have used evolving innovative designs to engage consumers and offer unique features and functions that could be translated to patient portal design. For instance, Apple’s ResearchKit’s Diabetes app pings the user daily to update disease and symptom-related information. Check-in questions or user-friendly alerts in portals could similarly be explored for engaging more patients their health care. Alerts could ask if the patient understands an abnormal result, direct them to helpful resources, and encourage test result follow-up. Finally, test results in the portal need to be easily understood by laypeople or displayed using simplified medical terms. For example, a portal might display elevated cholesterol as “↑LDL cholesterol,” or even just display the number without a flag, whereas a health app may label it as “bad cholesterol.”

In addition to literacy issues, patients want to know how a test result will affect them and whether or not they need to seek further care after receiving a test result. A notification accompanying the result with this information would be helpful for patients. For example, “Your result is slightly outside of the normal range. No further tests are needed; however, monitor your diet and cut back on high cholesterol foods listed on the American Heart Association website (link provided).”

While in traditional systems, physicians often include information explaining test results, current methods of automated direct release of test results to patients make this personalized note difficult. A notification system that focuses on how to relay this contextual information may help ease patients’ concerns and anxiety.

To increase use, user-experience, adoption, and functionality, patient portals need to incorporate innovative design and user-friendly features, such as simplified data displays, easy log-in access, and alerts, pings, or notifications that explain results in layman’s terms and tell the patient if additional care is needed. Many of these features coupled with ubiquitous access made possible by portable digital devices such as smart phones, wearable technology, and tablets, contribute to the increased use of mHealth apps. Moreover, mHealth apps may provide a sense of control to patients because of fewer barriers to login and options related to health or disease tracking, which patient portals do not possess.^42^ Future portal development could look towards mHealth apps in design, function, and user interface.^19^ Adding new features to portals may improve their use and encourage patients to track their overall health and disease states.

To encourage improved features and adoption, vendors will need to work on usability and design. There is now a growing demand for improving patient-centered care and communication.^43^ Additionally, the push for reimbursement that relates to value-based care creates an opportunity to develop high-quality patient portals. For example, getting a better understanding of patient outcomes beyond traditional measures collected by the health care setting (length of stay or 30 day read-mission rate) requires new methods of data collection from patients, such as their performance on activities of daily living.^44^ New functional requirements will require existing patient portals to improve their capability and usability if they expect to capture new measures, such as observations of daily living (ODL) data, or items to measure patient-centered communication in a meaningful way.^6^

Adoption of portals may increase if certain barriers related to internal organizational policies, procedures, culture, and environment as well as external rules, regulations, and pressures are overcome. For example, Table 1 lists patient portal barriers, such as an organization’s internal policies related to timely test result notification to patients and difficult or complicated sign up procedures that result from meeting HIPAA requirements for user authentication. These issues may be resolved with changes to external regulations and meaningful use requirements that encourage easy log-in and registration procedures. Further, continued iterative usability testing with both users and nonusers will help vendors and health care systems identify problems.

There is considerable variation among the major vendors on addressing some of the portal issues discussed herein. However, at least some vendors appear to be actively working to identify usability and safety issues that could make portals more patient-centric.^45^ With the recent relaxation of certain patient access parameters in meaningful use regulations, patients and patient-advocates have strengthened their call for better access to more meaningful health care data. Currently, most vendors do not allow third party development on top of patient portals; however, with the advent of the new Substitutable Medical Apps & Reusable Technology (SMART) on Fast Healthcare Interoperability Resources (FIHR)^46^ standards, we may see more third party app development in both the EHR and the patient portal. Once these new (albeit evolving) standards make their way into routine use, user interface design of patient portals should improve as a result.

It appears that the excitement over mHealth apps has likely begun to influence patient portal developers. In June 2014, Apple announced the HealthKit cloud application programming interface (API) and its partnership with Epic (Verona, WI), an electronic health record vendor who also makes MyChart (a popular patient portal), and the Mayo Clinic (Rochester, MN). Apple’s HealthKit cloud service collects and logs data that has been recorded by multiple sensors, apps, or monitors, like the accelerometer in the iPhone, and allows this information to be stored in a database or health profile.^47^ Information from multiple sources will be available in one source, and Apple and its partners are working on allowing health profile data to interact with the Epic electronic health record.^48^ If such systems are tested with patients to help further identify patients’ needs when viewing and interpreting health data, the engagement potential would likely increase.

## 6. Conclusion

Apps appear to have certain features that lead to better engagement success with patients, and this could potentially inform portal development. To improve user experience with future portals, developers could look towards apps in design, function, and user interface. Combining certain high-yield features of mHealth apps with the wealth of provider-generated data available in portals may improve portal use, increase patient engagement, and empower patients to track their health and disease(s). Nevertheless, continued research is necessary to understand how best to combine these features and how data can be used meaningfully by patients to improve outcomes. For further progress, informatics and human factors researchers will need to work in coordination with mHealth vendors, health care delivery organizations, and their data to determine how patients are using these health IT tools and how to make them most useful for patient care. This type of evidence is essential for creating value for patients, clinicians, and health care organizations, as well as for initiating changes to improve the patient portal. Both these health IT tools should be subjected to rigorous evaluation to ensure they meet their potential in improving patient outcomes.

## Figures and Tables

**Table 1:** Comparison of patient portals and health apps on eight socio-technical dimensions.

Dimension	Patient Portals	Health Applications
Hardware and Software	Accessible via computers, smart phones, and tablets Data is entered in system by labs and physicians	Accessible via computers, smart phones, and tablets Data is entered by consumers and imported via tracking devices
Clinical Content	Patients can access their personal health information (i.e., test results, immunizations) Direct Messaging with physician and health care team Contain medical terminology and acronyms that are unfamiliar to most patients^40^	Consumers can enter health information Data taken/entered in real-time Data is taken in from the tracking devices (i.e., the accelerometer in the iPhone) Generally no access to test results from physician No communication access to physician or health care team
Human-Computer Interface	Accessible from the web and smartphones Information is not always displayed in an understandable way, specifically test results Outdated user interface design	Simple to access, use, and navigate Information is often displayed in a way consumers without comprehensive medical knowledge can understand Up-to-date, simple user interface design
People	Aim to connect patients to information from health care system	Consumers can often connect and compete with other patients
Workflow and Communication	Patients sign up through their doctor’s office Patient password reset issues (e.g., in recent interviews, patients have complained about being “locked” out of their portal)	Consumers download the app and create their own account Consumers’ activities are passively tracked, reducing data entry Consumers have easy access to their data anytime, anywhere
Internal organizational policies, procedures, culture, and environment	Subject to an organization’s internal policies and procedures, which often create barriers to use (e.g., difficult sign-up procedures; reluctance of clinicians to participate in un-compensated work)	Currently subject to very little internal or external oversight (e.g., App developers can “sell” patient data) Culture is “move fast, fix problems later” Some have been found to sacrifice quality or safety in the pursuit of functionality^22^
External Rules, Regulations, Pressures	Must be HIPAA compliant Must meet the legal and confidentiality needs of adolescents^41^	HIPAA compliance under review Accessible to anyone with a smartphone Constantly evolving smartphone operating system requirements
System Measurement and Monitoring	Few organizations monitor or measure how patient portal information is being used Lack of real-time notifications and alerts to patients	Consumers use apps to monitor their own health Depending on the app, different alerts are sent to the consumer’s phone
